# High-Throughput Microfluidic Platform for 3D Cultures of Mesenchymal Stem Cells, Towards Engineering Developmental Processes

**DOI:** 10.1038/srep10288

**Published:** 2015-05-18

**Authors:** Paola Occhetta, Matteo Centola, Beatrice Tonnarelli, Alberto Redaelli, Ivan Martin, Marco Rasponi

**Affiliations:** 1 Department of Electronics, Information and Bioengineering, Politecnico di Milano, Milano, Italy; 2 Departments of Surgery and of Biomedicine, University Hospital Basel, University of Basel, Basel, Switzerland

## Abstract

The development of *in vitro* models to screen the effect of different concentrations, combinations and temporal sequences of morpho-regulatory factors on stem/progenitor cells is crucial to investigate and possibly recapitulate developmental processes with adult cells. Here, we designed and validated a microfluidic platform to (i) allow cellular condensation, (ii) culture 3D micromasses of human bone marrow-derived mesenchymal stromal cells (hBM-MSCs) under continuous flow perfusion, and (ii) deliver defined concentrations of morphogens to specific culture units. Condensation of hBM-MSCs was obtained within 3 hours, generating micromasses in uniform sizes (56.2 ± 3.9 μm). As compared to traditional macromass pellet cultures, exposure to morphogens involved in the first phases of embryonic limb development (i.e. Wnt and FGF pathways) yielded more uniform cell response throughout the 3D structures of perfused micromasses (PMMs), and a 34-fold higher percentage of proliferating cells at day 7. The use of a logarithmic serial dilution generator allowed to identify an unexpected concentration of TGFβ3 (0.1 ng/ml) permissive to hBM-MSCs proliferation and inductive to chondrogenesis. This proof-of-principle study supports the described microfluidic system as a tool to investigate processes involved in mesenchymal progenitor cells differentiation, towards a ‘developmental engineering’ approach for skeletal tissue regeneration.

The *in vitro* recapitulation of key mechanisms and temporal sequence of events involved in embryonic organogenesis is increasingly being recognized of great importance in the field of Tissue Engineering^1^ and more in general of regenerative medicine^2^,^3^. Several techniques for regenerating functional tissues have indeed found inspiration from developmental biology paradigms^4^, giving rise to the “so-called” field of developmental engineering^5^. In the context of skeletal tissues, this approach inspired the use of embryonic stem cells^6^ or human adult bone marrow-derived mesenchymal stem/stromal cells (hBM-MSCs)^7^,^8^,^9^,^10^ to recapitulate endochondral ossification processes through the early stages of limb development - namely cell condensation, undifferentiated proliferation of a mesenchymal cell population and pre-chondrogenesis. During development, these steps are tightly regulated by the interplay of specific signaling pathways – namely, Wnt/β-catenin, FGF and TGFβ/BMP – defining complex and spatio-temporal gradients^11^. In detail, the proper activation of Wnt-canonical and FGF pathways initially promotes the expansion of an undifferentiated pool of limb progenitors, which are subsequently capable to undergo chondrogenesis under the influence of members of the TGFβ/BMP superfamily^12^,^13^. Several studies have been carried out to elucidate the role of such pathways on hBM-MSCs fate, mainly using 2D cell cultures and, only recently, more relevant pellet-based 3D models^9^,^14^,^15^,^16^. However, these 3D approaches still suffer from an overall heterogeneity in cell responses and a consistently low proliferation rate^17^. Their inadequacy could be ascribed to (i) the non-physiological forced initial cell condensation, (ii) the presence of necrotic cores within the aggregates due to the high number of cells (typically ranging from tens to hundred-thousand cells), (iii) the suboptimal culture conditions (i.e. the poor control over morphogen delivery), and (iv) the formation of undesired chemical gradients within the volume of the samples due to diffusion limitations. More effective and reliable i*n vitro* models are thus required for investigating the response of mesenchymal cell systems to external morphoregulatory stimuli.

Microfluidics has been increasingly applied for generating high-throughput cell culture models, featuring unprecedented spatio-temporal control over microenvironmental conditions^18^. The establishment of a highly-controlled continuous perfusion of culture medium within microchannels has indeed been demonstrated to maintain more uniform and controlled culture conditions than traditional static approaches, providing constant convective dilution of catabolites and stable supply of nutrients and morphogenic factors^18^,^19^. Moreover, the ability to handle cells and fluids in precise configurations allows to tailor the microenvironment around cells, potentially achieving spatio-temporally controlled delivery of morphogen combinations^20^. Several microfluidic devices able to establish high-throughput 2D cell cultures were developed, either within research laboratories^21^,^22^,^23^,^24^ or as commercial platforms (e.g. CellASIC^TM^ ONIX Platform, Millipore). However, the control over the third dimension still remains poorly explored due to the challenge of combining microfabrication techniques with the size-scale of 3D micro-tissues. Although promising results have been accomplished^25^,^26^,^27^, the ability to combine generation, culture under continuous perfusion, and analyses of micro-tissues within a single microfluidic device, has only been achieved by means of self-aggregating embryoid bodies (EBs), and in low-throughput platforms^28^,^29^.

In this study, we combine the features of microfluidic and 3D culture systems with the goal of developing a more physiological *in vitro* model of limb development. To this aim, we report an innovative microfluidic platform for the generation and culture of 3D micromasses of adult hBM-MSCs under continuous and controlled laminar flow perfusion. The device consists of two functional units: a 3D culture area and a serial dilution generator (SDG). The culture area was specifically designed to favor the condensation of tens of mesenchymal cells within fluidically-connected microchambers located in spatially defined configurations, hence enabling the formation of micromasses with uniform size and shape. Two different SDG layouts were then implemented: the first characterized by a logarithmic configuration, allowing the investigation of soluble factors over a wide concentration range; the second featuring a linear layout for finer tunings within narrower concentration windows.

The developed microfluidic platform allowed us to achieve a more uniform and repeatable response of 3D perfused micromasses (PMMs) to specific morphogens involved in limb bud development (i.e. TGFβ, Wnt and FGF pathways), in comparison with traditional macroscale pellet culture models^14^. Finally, the effect of concentration patterns of a key morphoregulatory factor (TGFβ3) on hBM-MSCs 3D proliferation and differentiation was preliminarily investigated in a high-throughput fashion. This confirmed the suitability of the platform to be further exploited as an *in vitro* model to sequentially guide hBM-MSCs towards the primordial steps of the endochondral route.

## Results

### Microfluidic platform for generating and culturing 3D perfused micromasses

**Figure 1:**
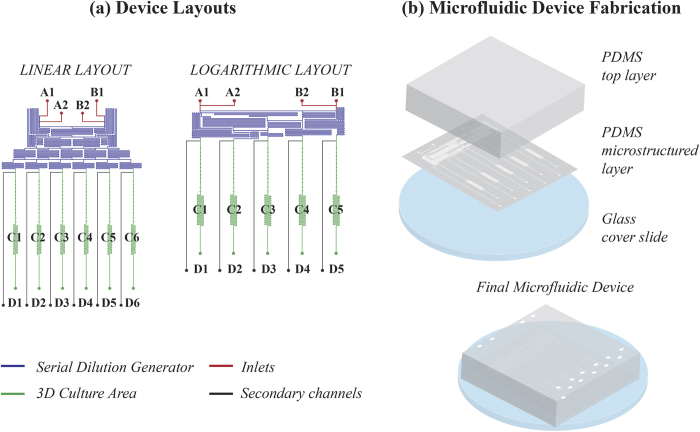
Microfluidic device layouts (**a**) and fabrication process (**b**) The chip layouts consist of a serial dilution generator – either featuring a linear or a logarithmic configuration - and a 3D culture area (**a**) Dilutions of chemicals can be generated from two main inlets (A1-B1) and delivered to downstream culture units (C1-C6); furthermore, each unit is provided with a secondary channel (D1-D6). Two additional inlets (A2-B2) facilitate the medium change operations, defining a by-pass for the device. The final device is fabricated with PDMS following standard soft-lithography techniques. First, the PDMS microstructured layer is bonded facing up to a PDMS top layer; then the device is bonded to a glass cover slide (**b**).

Two microfluidic platforms were designed for the generation and culture of cellular micromasses under spatially controlled chemical microenvironments (Fig. 1a). Two SDG layouts were designed based on a resistive flow scheme consisting of microfluidic networks^30^ able to generate linear (100%, 80%, 60%, 40%, 20%, 0%) or logarithmic (100%, 10%, 1%, 0.1%, 0%) concentration profiles of soluble factors. The microfluidic networks were composed of thin fluidic-resistance microchannels (70 μm wide and 70 μm high), whose lengths were dimensioned to allow diffusive mixing of soluble factors, while ensuring equal flow rates among the outlets. To this purpose, flow rates up to 12 μl/h were considered to mix chemical species characterized by diffusion coefficients as low as D ~ 10^-6^ cm^2^ sec^–1^. Both SDGs were rather compact, occupying a footprint of about 2.5 cm^2^. Due to the different design scheme, the required ratio between flow rates at input ports (A1 and B1 in Fig. 1a) were 1:1 and 1:3.5 for linear and logarithmic configurations, respectively.

Serial dilutions are independently delivered to downstream culture units. Each unit (Fig. 1a, C1-C6) comprises 10 cubic culture chambers (side of 150 μm) and it is integrated with a secondary channel (Fig. 1a, D1-D6). The chambers were dimensioned to culture 3D PMMs while ensuring enough space for their early expansion. Two secondary inlets (Fig. 1a, A2-B2) were also included in the layouts to facilitate the medium change operations, defining a by-pass for the device.

### Characterization of serial dilution generators

Linear and logarithmic SDGs were experimentally characterized in terms of distribution of flow rates and generation of designed chemical concentration patterns at the outlets.

Flow rates were rather uniformly distributed among the outlets in both SDG configurations. In the linear version, a total inflow of 24 μl/h was used (12 μl/h at each input port) and a maximum variation of 3% from the expected value (4 μl/h) was detected. Similarly, in the logarithmic configuration, a total inflow of 45 μl/h was imposed (10 μl/h at inlet A1 and 35 μl/h at inlet B1), resulting in a maximum variation of 5% from the expected outflow value (9 μl/h).

**Figure 2:**
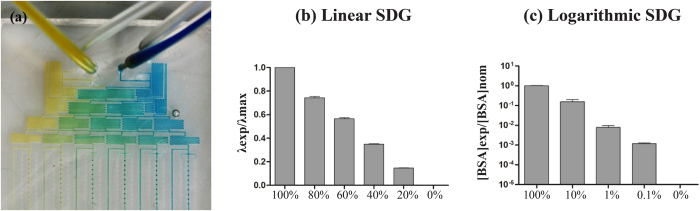
Characterization of serial dilution generators (SDGs). The linear device was perfused with dye solutions and, after reaching the steady state, a linear color pattern was visible in the SDG (**a**) The quantitative measurement of the emission wavelengths of the 6-outlet solutions (normalized for the λmax) resulted in a linear trend (n = 3) (**b**) The logarithmic device was characterized by perfusing 20 mg/ml BSA solution and measuring the concentration achieved after mixing within the SDG by means of a BCA assay. The quantitative measurement of the 5-outlet solutions resulted in a logarithmic trend, matching the expected concentrations (n = 3) (**c**).

The actual pattern profile of the chemical concentration was then assessed using two different readout approaches. For the linear SDG, two dye solutions were perfused at 10 μl/h and allowed to mix within the device, as exemplified in Fig. 2a. The six dilutions collected from the outlets matched with the expected linear distribution (R^2^ = 0.9891) (Fig. 2b). For the logarithmic SDG a more sensitive readout was required, being the output dilutions widespread over four orders of magnitude. Bovine serum albumin (BSA, Sigma, Buchs SG, Switzerland) protein was dissolved in distilled water (dH_2_O) and used for quantitative assessments. The BSA concentration of the five dilutions collected from the output ports were measured to range over four orders of magnitude (1, 0.1, 0.01, 0.001 and 0, as negative control), matching the expected logarithmic distribution (R^2^ = 0.9908) (Fig. 2c).

### Perfused micromasses generation within the microfluidic device

**Figure 3:**
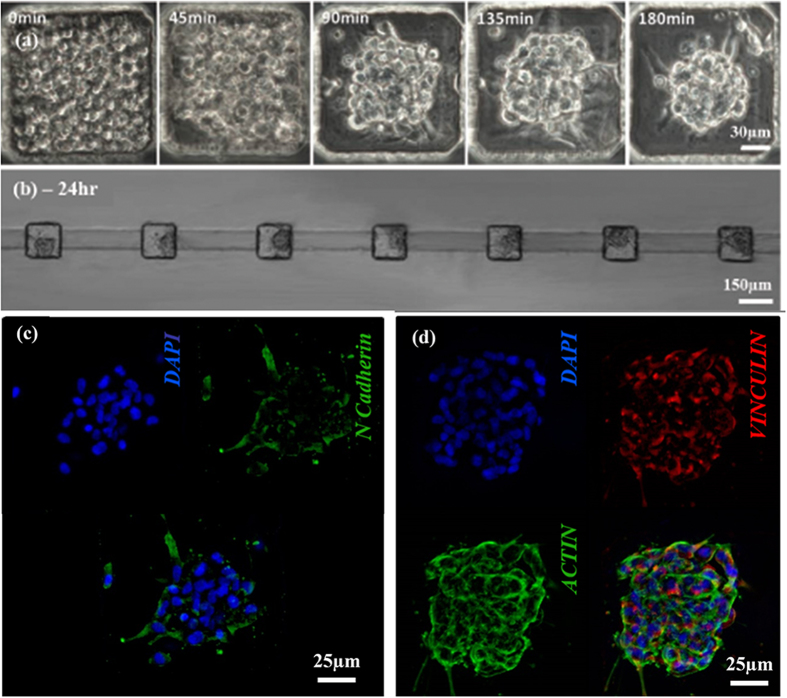
PMMs condensation. Time lapse phase contrast images of hBM-MSCs, initially filling a microchamber. Cells started to aggregate within tens of minutes while reaching a complete non-externally induced condensation after 3 hours from the seeding (**a**) Resulting PMMs were cultured within the chambers initially occupying about 3% of the volume (**b**) Immunofluorescence images showed the expression of adhesion proteins. The achievement of a full hBM-MSCs condensation is confirmed after 3 hours from seeding by N-cadherin expression, a cell adhesion molecule involved in the initiation of cell condensation (**c**) Moreover, the presence of an actin cytoskeleton network was showed, tightly connecting the cells after 3 hours from seeding (**d**).

Live phase contrast images of hBM-MSCs, initially inoculated into the microchambers, documented a progressive cell condensation (Fig. 3a). The condensation process was completed in about three hours from seeding, determining the formation of roughly spherical micromasses (Fig. 3b). To avoid culture medium evaporation during the cell condensation phase, a mild perfusion (500 nl/h) was maintained throughout this timeframe. At this time point, the micromasses featured a diameter of 56.2 ± 3.9 μm, occupying around 3% of the chamber volume, and consisting of 77 ± 15 cells, as predicted by experimental correlation based on a model described in Supplementary Information (SI1).

Early formation of adhesion contacts among cells was also assessed. Fig. 3c shows that neural cadherin (N-cadherin) started to be expressed within 3 hours after seeding, suggesting the initiation of cell-cell contacts. Noteworthy, the remodeling of actin cytoskeleton network as well as the expression of vinculin were also detected (Fig. 3d) within the interconnecting cells.

### 3D cell proliferation: comparison between macroscale and microfluidic models

Cell proliferation within PMMs was assessed over a period of 7 days^17^ in culture upon TGFβ3 (Transforming Growth Factor β 3) conditioning, and it was compared to traditional 3D macroscale cultures (macromass pellets). In particular, two parameters were monitored throughout the culture: (i) the total number of proliferative (i.e. Edu positive, Edu^+^) cells within samples, and (ii) the variations of aggregate dimensions.

**Figure 4:**
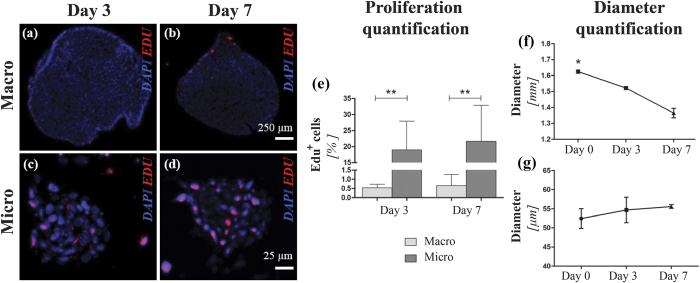
Comparison between macroscale pellet and microfluidic models in terms of 3D cell proliferation potential. Representative Edu immunofluorescence images showed a higher and more homogeneously spatially distributed number of Edu-expressing cells within the PMMs (**c**,**d**) than within the pellet sections (**a**,**b**) as confirmed by a statistically significant difference in the %Edu^+^ cells for both the considered time points (**e**) The assessment of the aggregates diameter over 7 days showed opposite trends: a statistically significant decrease for macroscale pellets (**f**) while a slight increase for the microfluidic model (**g**) (n = 3. For the microscale two donors were considered twice.*p *<* 0.05. **p *<* *0.01*).

Figure 4a,b and Fig. 4c,d show representative images of the Edu incorporation assay for both macro- and micro-aggregates, respectively. The macromass pellets exhibited up to 0.64 ± 0.34% Edu^+^ cells at day 7 (Fig. 4e), whereas a statistically significant higher number (i.e., 21.64 ± 11.13% at day 7, Fig. 4e) was found within PMMs. Noteworthy, Edu^+^ cells were mostly found along the contour of macromass pellets (Fig. 4a,b), while proliferative cells were uniformly distributed within the PMM volume (Fig. 4c,d). The specificity of the Edu incorporation was confirmed by analyzing colchicine-treated samples, where no proliferative cells were detected (see Supplementary Information, Fig. SI5d). Consistently, macromass pellets exhibited a statistically significant reduction in diameter (−15.3% ± 3.9%) from day 0 to day 7 (Fig. 4f), associated with a drop in the DNA content (see SI2). An overtime increase in dimensions was conversely measured for the PMMs (+4.7% ± 1.8%) during the same timeframe (Fig. 4g), correlated with an increase in cell number (see Supplementary Information, Fig. SI1).

### Effect of Wnt3a, FGF2 and TGFβ3 on perfused micromasses proliferation

The effect of different morphogen combinations on hBM-MSCs PMMs proliferation was assessed within the microfluidic platform.

**Figure 5:**
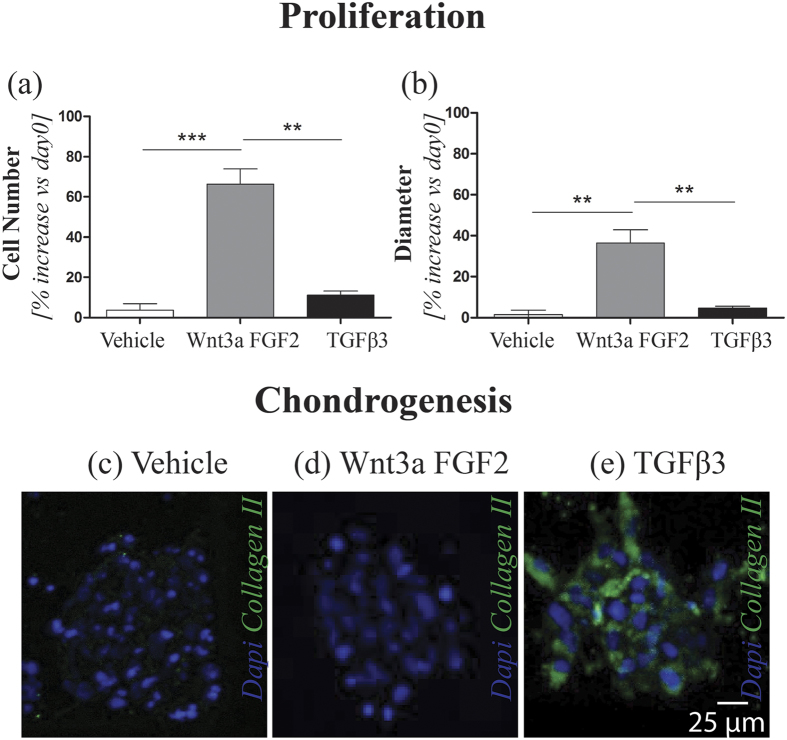
Effect of Wnt3a, FGF2 and TGFβ3 on hBM-MSCs microaggregates behavior. After 3 days in culture, both the percentage increase of cell number (**a**) and diameter (**b**) with respect to day 0 was significantly higher when microaggregates were conditioned with Wnt3a + FGF2 compared to TGFβ3 condition (**p *<* 0.01; ***p *<* *0.001)*. A negative control was also performed (named vehicle), culturing microaggregates in SFM without morphogens, leading during the 3 days culture to slight increases in cell number and microaggregate diameter, comparable with those obtained in the TGFβ3 group. Collagen II expression was then investigated for the three conditions. Results showed an expression of Collagen II only in the TGFβ3-treated group, possibly indicating its role in triggering a differentiation towards the chondrogenic lineage (**e**) Conversely, no Collagen II was detected neither in the (Wnt3a + FGF2)-stimulated group (**d**) or in the control condition (**c**).

Preliminary experiments were carried out on 2D cultures to identify a dose-response curve for different combinations and concentrations of morphogens on hBM-MSCs proliferation, yielding to 2D dose response curves. Based on the obtained results (Fig. SI3 and SI4), the most promising conditions in terms of cell proliferation were selected and translated within the proposed microfluidic model. In details, PMMs were cultured for 3 days in serum free medium (SFM) conditioned either with a combination of 20 ng/ml rhWnt3a (wingless-type MMTV integration site family member3a; R&D) and 5 ng/ml FGF2 (Fibroblast Growth Factor2) or with 1 ng/ml of TGFβ3 (Transforming Growth Factor β 3). A control condition (named vehicle) was also established by culturing PMMs in SFM only. After 3 days in culture, the vehicle condition exhibited slight increases in both cell number and microaggregate diameter, comparable with those obtained in the TGFβ3-treated group. However, the percentage increase of cell number with respect to day 0 was significantly higher when micromasses were perfused with Wnt3a+FGF2 (66.4% ± 15.1%) as compared to both TGFβ3 (11.1% ± 4.0%) and vehicle conditions (3.7% ± 6.4%) (Fig. 5a). Similarly, a statistically significant increase in micromasses diameter was also detected in the Wnt3a+FGF2-treated group (36.5% ± 12.8%) as compared to both TGFβ3 (4.7% ± 1.8%) and vehicle conditions (1.6% ± 1.7%) (Fig. 5b). These results confirmed the outcomes of the preliminary 2D experiments, underlining the preponderant role of Wnt3a and FGF2 in the enhancement of hBM-MSCs proliferation.

Type II collagen expression was then assessed for the three tested conditions, as to investigate the role of selected morphogens in the early expression of a chondrogenic marker. Results depicted in Fig. 5 show that type II collagen was only detected in the TGFβ3-treated group (Fig. 5e). Conversely, no collagen II was expressed either in the (Wnt3a+FGF2)-stimulated group (Fig. 5d) or in the control condition (Fig. 5c), confirming the different role of the tested morphogens in conditioning hBM-MSCs.

### Logarithmic screening of the TGFβ3 effect on perfused micromasses proliferation and differentiation

**Figure 6:**
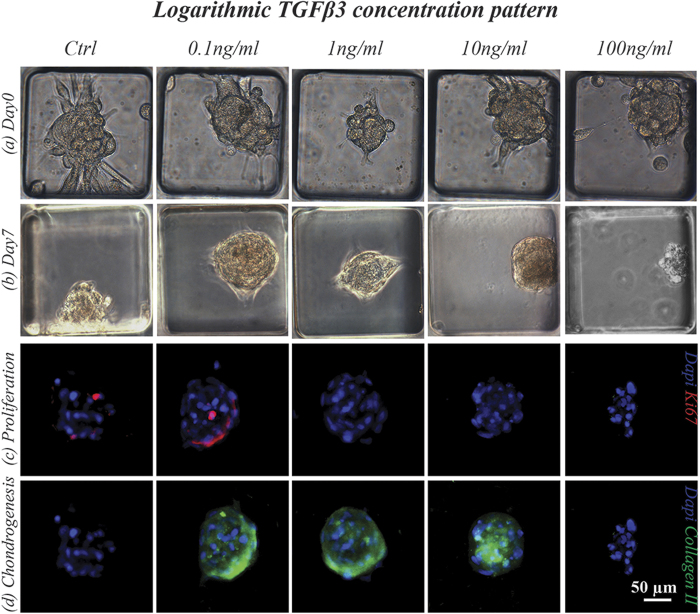
Logarithmic concentration screening over the effect of TGFβ3 on PMMs proliferation and differentiation. hBM-MSCs were cultured for 7 days under continuous perfusion of chondrogenic medium exhibiting a concentration gradient of TGFβ3 spanning over four order of magnitude (0, 0.1, 1, 10 and 100 ng/ml). Phase contrast images at day0 (**a**) and day7 (**b**) showed the toxic effect of the highest factor concentration, leading at micromasses disaggregation at the end of the culture period. Cell proliferation (**c**) and chondrogenesis (**d**) were investigated after 7 days in culture by staining PMMs for Ki67 and Collagen II expression, underling TGFβ3 concentration-dependent cell responses.

The microfluidic platform was finally exploited as a high-throughput tool for screening the concentration-dependent effect of morphogens on PMMs. By means of the logarithmic SDG device, four orders of magnitude of TGFβ3 concentrations were spanned (0.1, 1, 10 and 100 ng/ml, using 0 ng/ml as negative control condition) (Fig. 6). The highest concentration of TGFβ3 (100 ng/ml) led to micromasses disaggregation (Fig. 6a,b), with no detectable cell proliferation or chondrogenesis (Fig. 6c,d). At the concentrations commonly used for *in vitro* chondrogenic differentiation protocols^14^,^31^, cells did not proliferate but consistently deposited type II collagen. Interestingly, the lowest TGFβ3 concentration (0.1 ng/ml) showed to be able to maintain proliferating cells over seven days while inducing chondrogenic differentiation, as assessed by type II collagen deposition.

## Discussion

The recapitulation of the events occurring during limb development using adult stem/progenitor cells is currently being widely pursued in order to develop *in vitro* and *in vivo* strategies for skeletal tissue regeneration, according to the principles of “developmental engineering”^8^. In this regard, the reproducible instruction of clinically-relevant cell sources (e.g. hBM-MSCs) toward the endochondral route requires a deep understanding over their response to key morphoregulatory stimuli in 3D models^9^. In this study, we developed a platform for *in vitro* generation and culture of 3D mesenchymal cell micromasses under continuous and controlled perfusion, enabling a high-throughput exposure to exogenous factors and live cell imaging. We demonstrated that hBM-MSCs can form 3D aggregates upon injection into culture microchambers, and respond more uniformly and efficiently to key morphogens when perfused into the system as compared to traditional static approaches. The work thus provides a proof-of-principle of the microfluidic platform potentialities as a high-throughput tool to screen the effect of key morphogens in a 3D spatiotemporally-controlled fashion.

To date, several strategies have been developed to induce the condensation of cell micromasses by means of static approaches (i.e. hanging-drop techniques, microwells)^32^,^33^,^34^,^35^,^36^, aiming at demonstrating the benefits of the scale reduction of 3D models. Although effective, these approaches still rely on static and manual culture systems, unable to efficiently investigate 3D cell responses in an automated manner. Alternatively, pre-formed micromasses have been injected within microfluidic platforms for the subsequent culture under controlled conditions^25^,^26^,^27^. However, only few available devices can integrate both functionalities, and they mainly rely on the intrinsic ability of embryonic cells to spontaneously form EBs^28^,^29^. Attempts have been recently made to generate microaggregates within microfluidic platforms starting from adult cell sources; nonetheless, they were mostly based on complex multiple-layer designs in which cell condensation was induced through the formation of vortices and rotational flows within microchambers^37^,^38^.

We developed an innovative microfluidic platform consisting of a single-layered device integrating independent culture units, each featuring 10 cubic chambers (thus potentially useful for studying the effect of paracrine factors), serially connected to a fluidic network designed to precisely deliver given concentrations of morphogens. Such powerful microfluidic tool integrates for the first time the capabilities to (i) uniformly generate 3D cell micromasses into defined spatial configurations and (ii) culture them under continuous laminar flow perfusion of specific morphogens concentrations. In particular, the platform allowed obtaining condensation of tens of hBM-MSCs (77 ± 15) within each culture microchamber into spherical micromasses, without the need of any rotational flow or external induction. Through the use of a computational fluid-dynamics model, the shear stress experienced by hBM-MSCs in this phase was indeed estimated to be negligible to cell condensation^39^ (see Supplementary Information FigSI6). The condensation phenomenon, hence classified as non-externally induced, was possibly due to (i) the low adhesiveness of the PDMS substrate, (ii) the geometry and size of the microwells, and (iii) the relative ratio between the cell number and well size, yielding a high cell density in a confined small volume. The non-externally induced condensation of hBM-MSCs is an important achievement in comparison with the traditional scaffold-free systems involving 3D mesenchymal cell cultures, which are mainly based on macromass pellets obtained by centrifugation^14^,^17^. This initial forced centrifugation step indeed leads to the establishment of too tight cell-cell and cell-ECM interactions, high variability in the time required to form 3D aggregates (24÷72 hours), and non-uniformly shaped tissues^40^. Conversely, the proposed platform was permissive of an initial condensation of hBM-MSCs within 3 hours, generating micromasses featuring rather uniform sizes (56.2 ± 3.9 μm). The early organization of actin microfilaments in the cells coupled with a rather uniform expression of vinculin and N-cadherin suggests an effective and functional interconnection among cells within the PMMs. However, further investigations should be performed to (i) characterize the biological pathways involved in hBM-MSCs aggregation, (ii) study the temporal maturation of such complex network, and (iii) compare the cell condensation dynamics in the proposed model with those occurring during limb development.

The platform was further exploited to culture generated micromasses under controlled perfusion aiming at investigating the uniformity of their response to external morphogens stimuli. As compared to traditional macropellets models, which mainly suffered from an overall heterogeneity in cell responses correlated with the presence of necrotic cores^17^,^41^, PMMs exhibited a 33.8-fold higher percentage of Edu^+^ cells, homogeneously spread throughout the whole volume. These observations confirmed the potentiality of the model in replicating the 3D expansion step occurring during the early stages of limb development^11^, thus overcoming one of the main limitations of macroscale models, i.e. an overall drop in cell number regardless of the culture conditions^17^ (see supplementary information, fig. SI2).

Furthermore, a pre-chondrogenic expansion similar to that occurring in the limb bud was achieved. Indeed, through a combination of Wnt3a and FGF2, an increase in cell number of 66.4 ± 15.1% was observed in the PMMs from day 0 to day 3, whereas no expression of collagen type-II was detected. Conversely, the perfusion of TGFβ3-based medium induced an earlier onset of TGFβ-mediated chondrogenesis as compared to macroscale models^42^, as exemplified by the expression of collagen type-II already after 3 days in culture.

Finally, the use of the serial dilution generator unit allowed to screen the concentration-dependent effect of a key morphoregulatory factor (i.e. TGFβ3) on the 3D hBM-MSCs model. The obtained proof-of-principle results underline the efficacy of the presented model to detect different cell responses in the presence of different morphogen concentrations, spanning over four orders of magnitude. Interestingly, the lowest concentration tested (100 pg/ml) was sufficient to trigger specific cellular responses (i.e. chondrogenic specification and cellular proliferation), which cannot be detected either at the macroscale or in 2D models, without delivering at least hundred times higher concentrations of the morphogen. Our findings indicate the potentiality of the presented microfluidic system to investigate the effect of key morphogens, applied in a controlled and high-throughput fashion in different sequences, combinations and concentration ranges, on early stages of 3D chondrogenesis by hBM-MSCs. Moreover, the presence of secondary inlets within the platform will allow the integration of an automated control over the delivery of morphogens to further investigate time-dependent effects of such key signaling pathways.

In conclusion, our study suggests a possible use of such microfluidic device as *in vitro* platform for “development-inspired” investigations. After assessing the portability of the presented technology to different cell sources (e.g. mesenchymal limb progenitors), we envision the possibility to extend the applicability of this platform to different biological models, where initial cell condensation and the onset of a 3D structure is expected to mediate a physiological response to exogenous signals. Once clarified these critical points, the proposed microfluidic platform may find further applications in the field of developmental biology - e.g. as an *in vitro* limb bud model - as well as in the pharmaceutical industry for screening compounds intended to induce regeneration of mesenchymal tissues.

## Methods

All reagents were purchased from Gibco (Life Technologies, Zug, Switzerland) if not otherwise indicated.

### Ethics statement

Primary human cells were obtained from 3 healthy donors (24-44 years old, mean age: 34) during routine orthopedic surgical procedures, following informed patient consent and in accordance with the approved guidelines. The experimental protocol was approved by the local ethical committee (Ethische Kommission Beider Basel, EKBB, Ref. 78/07).

### Microfluidic platform fabrication

The microfluidic layouts (Fig. 1a) were designed by means of a CAD software (AutoCAD, Autodesk Inc.) and the corresponding master molds were realized through photolithography techniques^43^. A thin microfluidic layer (thickness 1 mm) was obtained by replica molding of polydimethylsiloxane (PDMS; Sylgard^®^ Dow Corning, Michigan, USA). Briefly, liquid PDMS was cast on the mold in ratio 10:1 w*/*w (pre-polymer to curing agent), degassed and cured at 80 °C for 3 h. The obtained PDMS microstructured layer was permanently bonded facing up to a flat PDMS slab (thickness 5 mm), upon 1 min of air plasma treatment (Harrick Plasma, NY, USA), thus closing the channels. Input and output ports were obtained through a 0.5 mm biopsy puncher (Harris Uni-Core^TM^). The chip was finally permanently bonded to a glass coverslide (diameter 50 mm, thickness 150 μm; VWR International Inc., PA, USA) upon an additional 1 min exposure to air plasma (Fig. 1b). External connections were realized through tygon tubings (ID = 0,02”; Qosina, NY, USA) and stainless steel couplers (23 gauge; Instech Laboratories Inc., PA, USA).

### Characterization of the serial dilution generator

Linear and logarithmic SDGs were characterized in terms of flow rate distribution to verify the correct partitioning of the inflow among culture units. Tygon tubings were connected at the output ports and an overall flow rate of 24 μl/h for the linear SDG and 45 μl/h for the logarithmic one was imposed through the inlets. The outflow from each outlet was quantified by measuring the travel length of each meniscus within the tubing.

Quantitative evaluations of chemical patterns generated by the SDGs were performed^44^. For the linear configuration, two dye solutions were pumped through the main inlets at a flow rate of 10 μl/h. After the achievement of a steady state condition, samples of 20 μl (n = 3) were collected from each outlet. A spectrophotometer (NanoDrop 2000c, Thermo Scientific, Wilmington, USA) was used to detect the average emission wavelength for each condition. For the logarithmic SDG, a solution of 20 mg/ml BSA in dH_2_O was pumped into the right inlet (A1) at a flow rate of 10 μl/h while dH_2_O was pumped into the left one (B1) at 35 μl/h. After the achievement of a steady state condition, samples of protein dilutions were collected from each outlet (n = 3) and the BSA concentration was measured by means of a BCA Protein Assay kit (Pierce, Thermo Fisher Scientific, Reinach, Switzerland), following the manufacturer’s indications.

### Cell isolation and expansion

Human mesenchymal stromal cells (hBM-MSCs) cultures were established as previously described^45^. hBM-MSCs were expanded in alpha-MEM containing 10% fetal bovine serum (FBS), 4.5 mg/ml D-glucose, 0.1 mM nonessential amino acids, 1 mM sodium pyruvate, 100 mM HEPES buffer, 100 Ul/ml penicillin, 100 μg/ml streptomycin, and 0.29 mg/ml L-glutamate, further supplemented with 5 ng/ml FGF2 (Fibroblast Growth Factor2; R&D Systems, Minneapolis, MN, USA). Medium was changed twice a week and hBM-MSCs were used between P1 and P3 for the experiments.

### Macroscale pellet cultures

Macromass pellets were established and used as control. In details, aliquots of 2.5 × 10^5^ cells/0.25 ml hBM-MSC were centrifuged at 1,100 rpm for 5 min to form pellets. Pellets were cultured under static conditions up to one week in SFM, consisting of DMEM with 1 mM sodium pyruvate, 100 mM HEPES buffer, 100 UI/ml penicillin, 100 μg/ml streptomycin, 0.29 mg/ml L-glutamate, 0.1 mM ascorbic acid 2-phospate (Sigma), ITS+1 (Sigma, 10 μg/ml insulin, 5.5 μg/ml transferrin, 5 ng/ml selenium), 5 μg/ml human serum albumin, and 4.7 μg/ml linoleic acid further enriched with 1 ng/ml TGFβ3 (Transforming Growth Factor β 3) and medium was changed twice a week.

### Perfused micromasses generation and culture within the microfluidic device

Before cell seeding, microfluidic devices were sterilized by autoclaving (121°C, 20 min, wet cycle) and dried overnight at 80°C to recover PDMS hydrophobicity and minimize cell adhesion in the chambers during seeding. Devices were then submerged in sterile PBS and degassed^46^. Subsequently, SFM was perfused through the main inlets of the device (Fig. 1a, A1 and B1).

After expansion hBM-MSCs were detached, diluted to the concentration of 10^7^ cells/mL in SFM, and seeded into the culture units by perfusing 0.1 μl/min of cell suspension from all outlets (C1-C6), while maintaining an opposite mild medium flow (0.5 μl/min) from each inlet (A1-B1). The secondary channels (D1-D6) were used as waste lines both during the seeding procedure, to avoid cell clogging in the upstream channels, and during the initial cell condensation phase, to reduce the shear stress experienced by seeded hBM-MSCs. Secondary channels were instead clamped during the culture period. Once the culture chambers were filled with hBM-MSCs, a continuous flow rate of 5 μl/h *per* line was applied and maintained throughout the entire experiment, while the microfluidic platform was incubated under standard culture conditions (T = 37°C, 5% CO_2_ and humidified atmosphere). Phase contrast images of hBM-MSCs seeded into the microchambers were acquired every 15 minutes during the first 4 hours of culture to monitor the micromasses condensation.

The generated micromasses were cultured up to seven days under continuous perfusion of different conditioning media. In details, SFM was enriched either with 1 ng/ml TGFβ3 (Transforming Growth Factor β 3) or with a combination of 20 ng/ml rhWnt3a (wingless-type MMTV integration site family member3a; R&D) and 5 ng/ml FGF2 (Fibroblast Growth Factor2).

### 3D cell proliferation assays

SFM enriched with 10 μM Edu was either added to the macropellet cultures or continuously perfused within the microfluidic device at specific time points (day 2 and 6). Different incubation times with Edu were tested (see Supplementary Information, Fig. SI5a-c) and an exposure time of 24 hours was selected as the one giving an adequate temporal window over the cell cycle^17^. The quantification of Edu^+^ cells was performed via immunofluorescence staining upon incorporation of Edu, as described in the *Immunofluorescence analyses* sub-section. Negative controls were obtained by treating samples with colchicine (100 ng/ml; Invitrogen, Life Technologies) from day 0 of culture, to inhibit cell division (see Supplementary Information, Fig. SI5d). Further negative controls were established by preventing cells from Edu exposure (see Supplementary Information, Fig. SI5e).

PMMs live phase contrast images were acquired using an Olympus BX-61 (Olympus, Tokyo, Japan) and the average diameters were evaluated by means of the ImageJ software ( http://imagej.nih.gov). The corresponding cell number was calculated in correlation with the micromasses diameter applying an algorithm developed by Martin *et al.*^47^ and validated for our specific samples (see Supplementary Information). Concerning macropellets, the diameter was directly measured, while the cell number quantification was carried out by means of a standard CyQUANT^®^ cell proliferation assay kit (Molecular Probes, Life Technologies).

### Logarithmic screening over the effect of TGFβ3 on micromasses

Exploiting the SDG unit, hBM-MSCs micromasses were cultured under continuous perfusion of chondrogenic medium (SFM enriched with of 10^-7^ M dexamethasone), while serial dilutions of TGFβ3 were established throughout the logarithmic SDG configuration. After 7 days in culture, PMMs were analyzed both in terms of cell proliferation and chondrogenic differentiation, by immunofluorescence for Ki67 and collagen II, respectively, as described in the specific sub-section.

### Immunofluorescence analyses

At specific time points, immunofluorescence analyses were performed using Dapi as counterstaining. Microaggregates were fixed by perfusing 4% paraformaldehyde for 20 min and immunofluorescence stainings were obtained by sequentially perfusing the required solutions directly within the device at constant flow rate (1 μl/min). Briefly, a solution of 3% goat serum and 0.5% Tween20 (Sigma) in PBS was perfused for 45 min to permeabilize cells and to block nonspecific bindings. Expression of vinculin and N-cadherin were detected by perfusing 10 μg/ml Mouse Anti-Vinculin (BD, Allschwil, Switzerland) and Mouse Anti-N cadherin (R&D) for 1 hour. Actin cytoskeleton was stained by further perfusing a FITC-conjugated phalloidin solution (BD) for 45 min. Cell proliferation and expression of collagen II within microaggregates was also evaluated by perfusing 10 μg/ml of Rabbit Anti-Ki67 and 10 μg/ml of Mouse Anti-Collagen II (R&D) solutions for 1 hour. Finally, microaggregates were perfused with specific secondary antibodies (Goat Anti-Mouse IgG1 Alexa Fluor 546-conjugated for vinculin, Goat Anti-Mouse IgG1 Alexa Fluor 488-conjugated for N-cadherin and collagen II and Goat Anti-Rabbit IgG for Ki67) for 45 min.

Immunofluorescence for Edu staining was performed on both Edu-treated PMMs and sections of macropellets by using Alexa Fluor 555-conjugated Click-iT Edu Assay kit (Molecular Probe, Life Technologies), according to the manufacturer’s indications. The micromass staining was performed by sequentially perfusing the required solutions directly within the microfluidic platform, as described above. Macropellets instead were fixed in 4% paraformaldehyde overnight, dehydrated, and embedded in paraffin. Five micrometers thick sections were then cut by means of a Microm HM400 microtome and the Edu staining was obtained following a standard protocol^17^.

Confocal images of immunofluorescence micromasses were acquired directly within the devices by means of a Nikon A1R Nala Confocal microscope (Nikon, Tokyo, Japan), while immunofluorescence sections of macropellets were analyzed by means of an Olympus BX-61 microscope^17^. The 3D percentage of Edu^+^ cells over the total number of cells was calculated through ImageJ software (NIH).

### Statistical analysis

Data are expressed as mean ± standard deviation, and statistical analyses (t-test and one-way ANOVA, with a 99% confidence level, Bonferroni post-hoc test) were performed using GraphPad Prism v5.00 (GraphPad Software, San Diego, CA).

## Additional Information

**How to cite this article**: Occhetta, P. *et al.* High-Throughput Microfluidic Platform for 3D Cultures of Mesenchymal Stem Cells, Towards Engineering Developmental Processes. *Sci. Rep.* **5**, 10288; doi: 10.1038/srep10288 (2015).

## Supplementary Material

Supporting Information
