# Supplementary material for: High-Throughput Microfluidic Platform for 3D Cultures of Mesenchymal Stem Cells, Towards Engineering Developmental Processes
Source: Sci Rep. 2015 May 18;5:10288. doi: 10.1038/srep10288 (PMC4650750; doi:10.1038/srep10288)
Supplement: Supporting Information [file srep10288-s1.pdf]

High-Throughput Microfluidic Platform for 3D Cultures of Mesenchymal Stem Cells, Towards Engineering Developmental Processes

Paola Occhetta[1,2,*], Matteo Centola[2,*], Beatrice Tonnarelli[2], Alberto Redaelli[1], Ivan Martin[2], Marco Rasponi[1]

Supplementary Information

Correlation between microaggregates diameter and cell number

A previously implemented algorithm [1] for determining the number of chondrocytes in an aggregate starting from its two-dimensional macroscopic profile was applied to our model. Briefly, the algorithm correlates the diameter of an aggregate with the corresponding cell number by means of a sphere packing theory. Fig.SI1 shows the theoretical relationship between diameter and cell number, having upper and lower bounds.

Experimental results obtained through our microfluidic approach were compared to the algorithm in order to determine the degree of matching. In details, hBM-MSC microaggregates were formed within the platform and after 3 hours live phase contrast images were acquired by means of a Olympus BX-61 microscope. The diameter of each microaggregate was calculated as the average of two measurements. Microaggregates were subsequently fixed in 4% PFA and immunofluorescence stained for Dapi as described in the Materials and Methods section. Confocal Z-stack images of the immunofluorescence microaggregates were acquired by means of a Nikon A1R Nala Confocal microscope. The number of cells was then calculated through the ImageJ software (NIH).

The experimental results obtained with hBM-MSCs microaggregates were shown to match with the theoretical model (Fig.SI1) with a high degree of fidelity, namely R^2=0.9565. The model was thus employed during the experiments for estimating the number of cells starting from the measurement of microaggregates diameter.

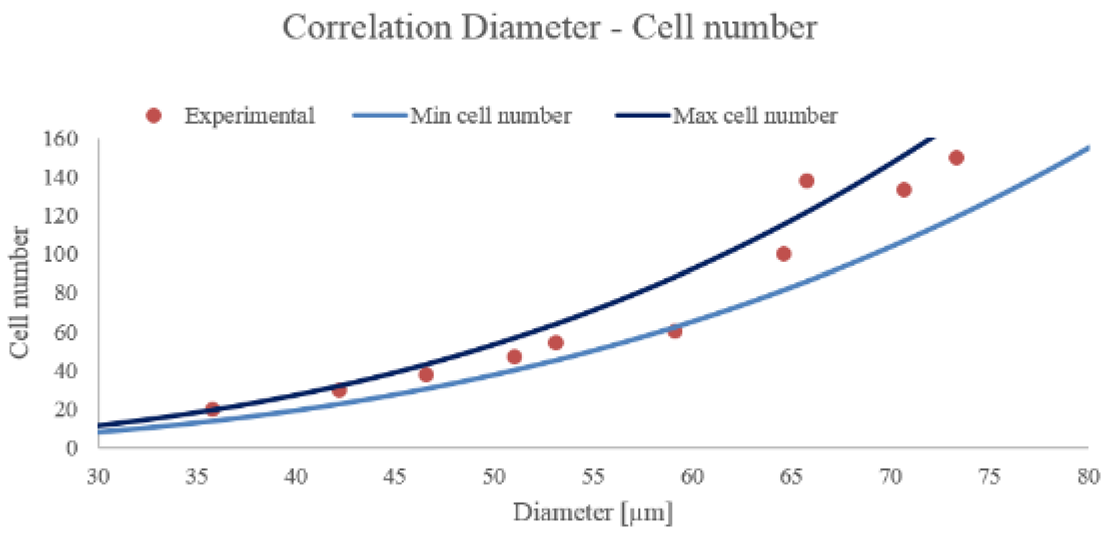

Fig.SI1 Validation of a previously implemented algorithm (Martin et al., 1997) for correlating diameter and cell number in the proposed hBM-MSCs microaggregates model. The theoretical relationship between diameter and cell number were plot and compared to the experimental data (a).

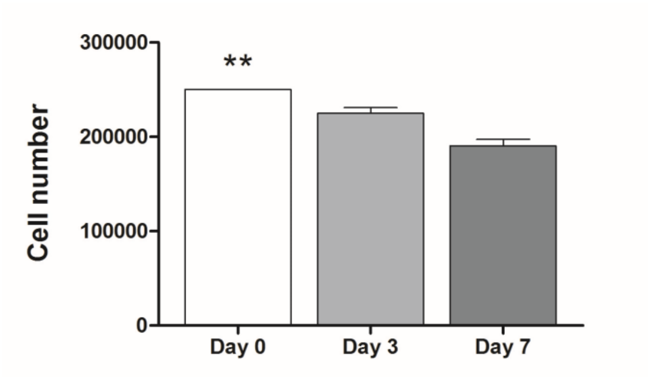

Fig.SI2 CyQuant quantification of cell number within macroscale pellets during 7 days of culture.

Wnt3a, FGF2 and TGFβ3 dose dependent study over 2D hBM-MSCs proliferation

Preliminary experiments were carried out on 2D hBM-MSCs cultures for screening the influence of different combinations and concentrations of morphogens on hBM-MSCs proliferation. In details, Wnt3a, FGF2 and TGFβ3 were considered and hBM-MSCs dose dependent 2D proliferation responses to those morphogens were assessed. Briefly, hBM-MSCs (n=4 donor) were cultured in monolayer (2D) in a 96-well plate at 3,000 cells/cm2 for 72h and exposed to different concentrations of Wnt3a (5–40 ng/ml), FGF2 (5–40ng/ml) and TGFβ3 (0.1-100 ng/ml). The effect of combinations of Wnt3a and FGF2 on hBM-MSCs proliferation was also assessed, as they are known to act synergistically during limb bud early expansion stages [2]. In details, the following combinations were tested: FGF2 5ng/ml and Wnt3a 20ng/ml; FGF2 20ng/ml and Wnt3a 5ng/ml; FGF2 5ng/ml and Wnt3a 5ng/ml; FGF2 20ng/ml and Wnt3a 20ng/ml. Finally, a negative control condition (named vehicle) was established, culturing cells in SFM only. Each condition was tested in triplicate.

At day 3, the total cell number was assessed for each condition by quantified the number of nuclei, stained with Dapi as previously described. The 96-well plate was read by using the Operetta High Throughput Imaging System (Perkin Elmer). A total of 45 fields/well were acquired with a 20X magnification objective. The total number of cells and the % of Edu+ cells (n=135, 45 fields per well, and three wells per condition) were calculated by means of the Columbus Image Data Storage and Analysis System (Perkin Elmer).

In Figures SI3 and SI4, hBM-MSCs 2D proliferative response to Wnt3a, FGF2 and TGFβ3 is normalized to the vehicle condition. Considering Wnt3a and FGF2 dose-responsive curves (Fig.SI3), the combination of FGF2 5ng/ml and Wnt3a 20ng/ml (red column in Fig.SI3) resulted in the highest cell number, double with respect to the vehicle control and comparable only with the 5ng/ml FGF2 condition, which is the traditional golden standard for maintaining 2D hBM-MSCs in an undifferentiated state [2]. These results suggested how these factors could play an orchestrated role in guiding hBM-MSCs proliferation, and for this reason, we decided to test their combined effect within the proposed microfluidic model.

Regarding the TGFβ3 dose-dependent effect on 2D hBM-MSCs proliferation (Fig.SI4), a slight increase in cell number was detected only considering 1 and 10 ng/ml, while the boundary concentrations (0.01 and 100 ng/ml) gave results comparable to the negative control. Moreover, analysis on cell morphology suggest a cytotoxic effect of this morphogen in all the concentration tested, inducing a change in hBM-MSCs morphology from elongated to cuboidal shapes. Nevertheless, being TGBβ3 known to be a key requirement for 3D hBM-MSCs chondrogenesis [3], we decided to test the dose-dependent effect of this morphogen in our microfluidic system.

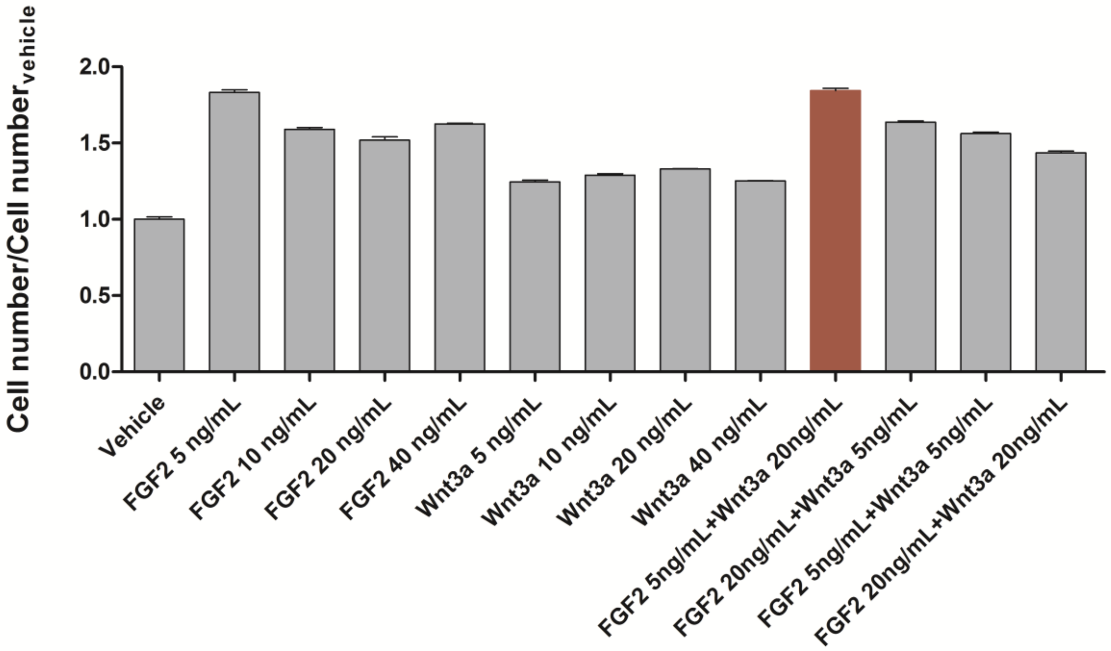

Fig.SI3 Dose-dependent effect of Wnt3a and FGF2 on 2D hBM-MSCs proliferation. The red column underlines the condition inducing the highest hBM-MSCs proliferative response, corresponding to the combination of FGF2 5ng/ml and Wnt3a 20ng/ml.

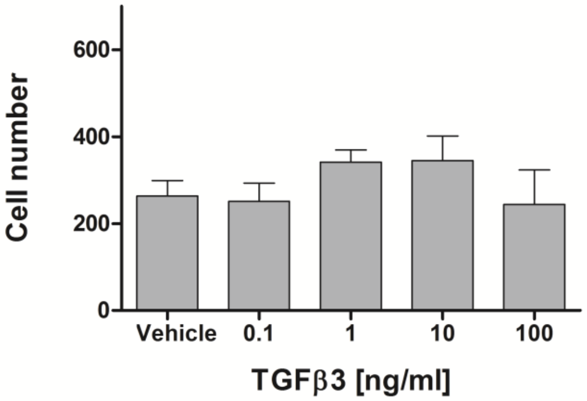

Fig.SI4 Logarithmic dose-dependent effect of TGFβ3 on 2D hBM-MSCs proliferation.

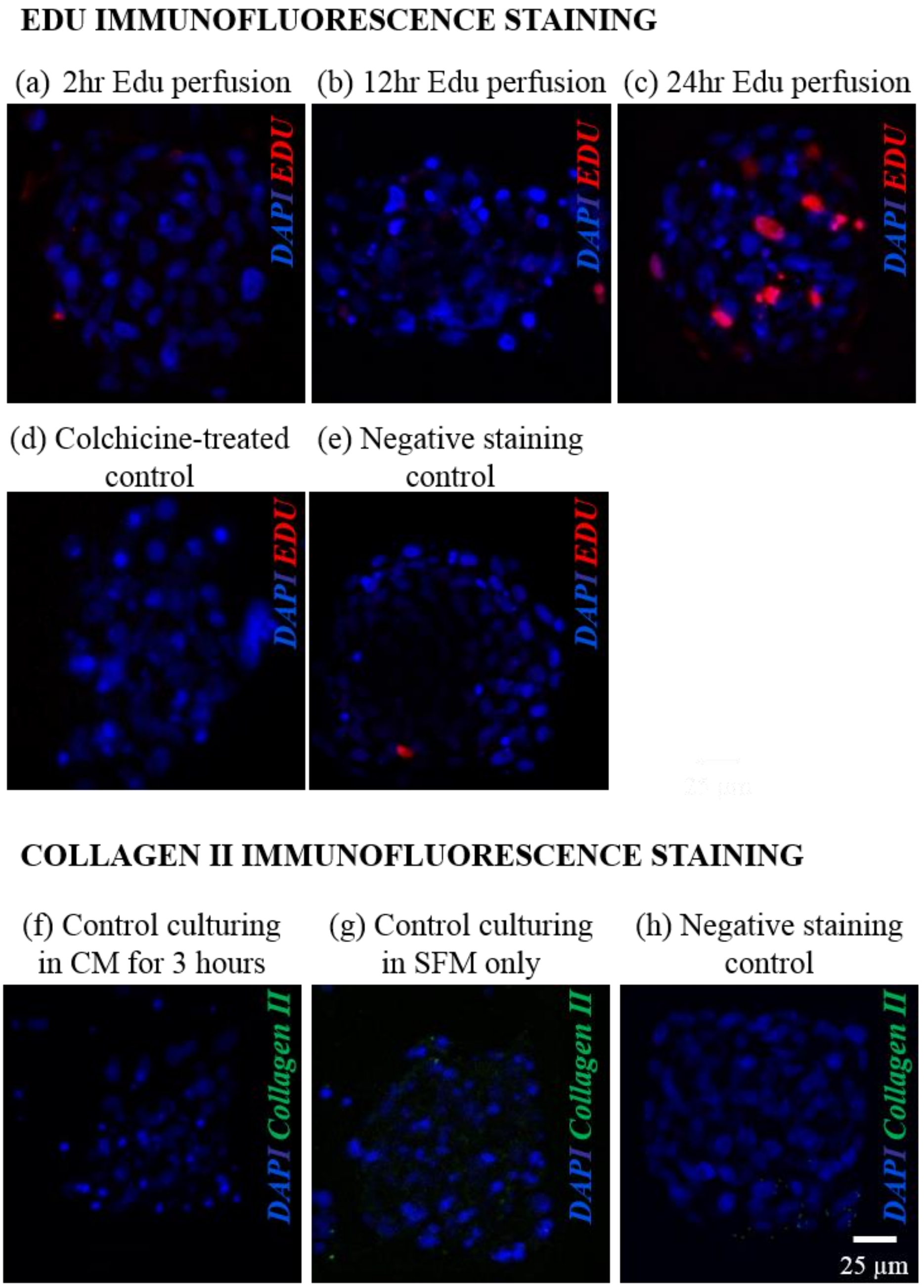

Fig.SI5 Controls defined for both the Edu and collagen II immunofluorescence stainings Different Edu exposure time were tested for studying the 3D proliferation of microaggregates (2hr, 12hr and 24hr) (a-c), obtaining adequate results in terms of temporal window over the cell cycle only for the 24hr exposure (c). To establish an experimental negative controls, some samples were treated with colchicine to inhibit cell division (d). Negative staining controls were finally obtained preventing cells from Edu exposure (e).

The baseline collagen II expression of hBM-MSC microaggregates was obtained after 3 hours of culture in chondrogenic medium (f). An experimental negative control was acquired after culturing the microaggregates in SFM (g). Negative staining controls were finally obtained preventing cells from primary antibody exposure (h).

Computational study over the role of perfusion during micromasses generation

The influence of perfusion on hBM-MSCs condensation was numerically assessed by means of Computational Fluid Dynamic modeling (CFD, Comsol Multiphysics). In detail, finite element analyses were performed on a 3D geometrical model of six cubic perfused microchamber (side $h_{chamber}$=150μm) perfused by a 100μm wide and 70μm high channel. Six different chamber layouts were conceived so as to model the entire range of filling conditions upon cell seeding. Such modeling was accomplished by progressively decreasing the height of the chamber, starting from the empty condition ($h/h_{chamber}$=100%) to that corresponding to a chamber completely filled with cells ($h/h_{chamber}$=50%). Geometries were discretized through a tethraedrical mesh scheme, consisting of about 2 million elements. The flow field was computed by solving stationary Navier–Stokes equations for incompressible fluids, with density and viscosity equal 1000 kg/m^3 and 0.890 cP, respectively [4]. A uniform velocity profile was applied to the inlet, corresponding to a total inflow of 0.5μL/h, estimated to be the perfusion rate experienced by cells during the initial condensation period within the device. A zero pressure condition was set to the outlet and a no-slip condition was applied to boundary walls. Equations were iteratively solved through the generalized minimal residual method (GMRES), and the convergence criterion was satisfied when all norm residuals fell below 1×10^{-6}.

Figure SI6a shows the computed velocity profiles characterizing the six considered conditions, while in Fig.SI6b shear stresses acting at the bottom surface of the chamber are provided as an estimate of the shear level experienced by seeded cells, both in terms of average and maximum values. In all conditions, even in the most critical scenario (i.e. in the case of completely full chamber), the shear stress due to the mild perfusion applied was lower than $2x10^{-2}$ dyn/cm^2.

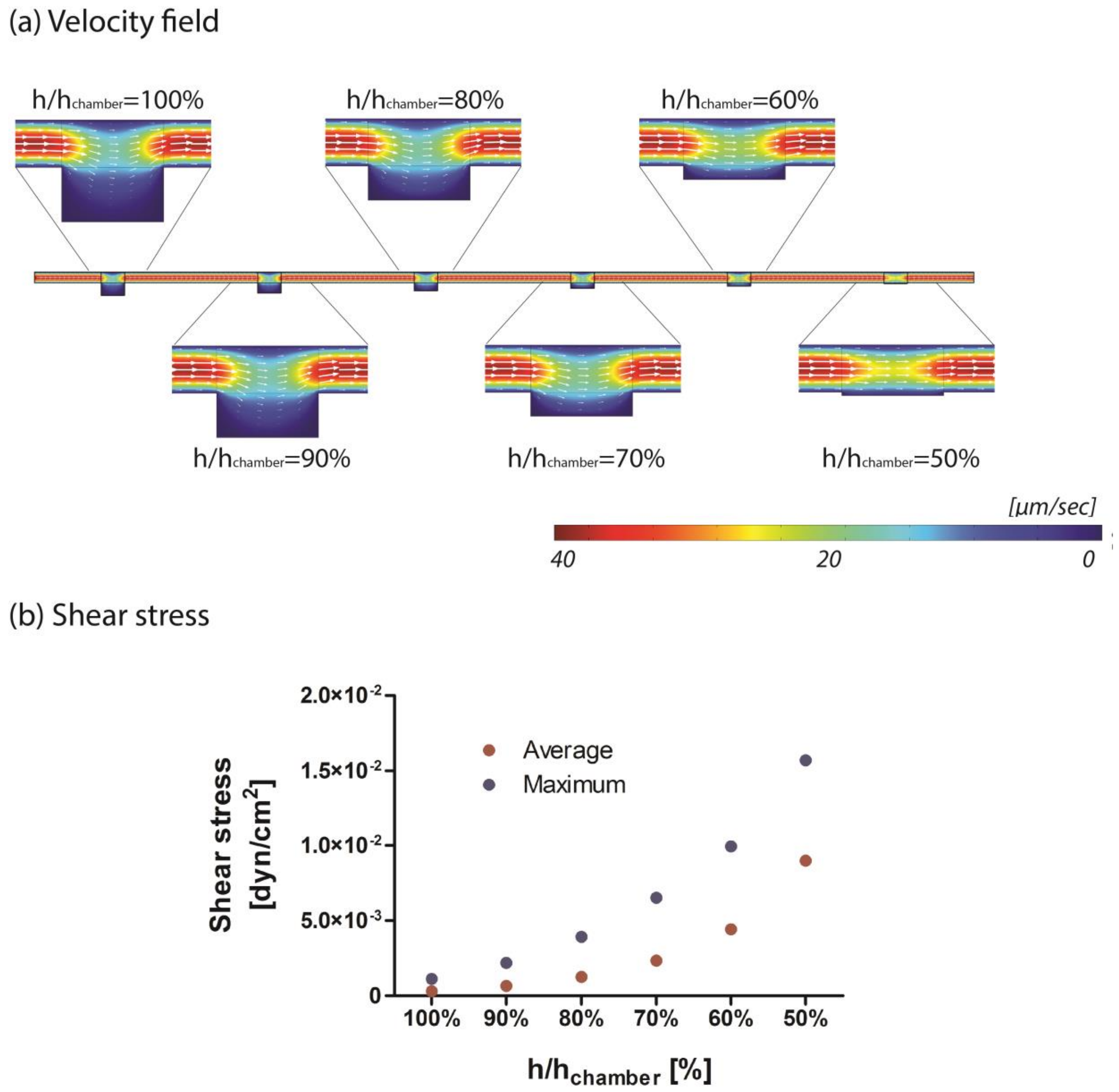

Fig.SI6 CFD computation of velocity fields experienced by cells trapped within culture chambers during the initial condensation (a). A perfusion flow rate of 0.5μl/h was considered and six different seeding conditions modeled, from an empty chamber ($h/h_{chamber}$=100%) to a completely filled one ($h/h_{chamber}$=50%). Maximum and average shear stress acting on seeded cells were then calculate for each condition tested (b).

Microaggregates viability assessment

To assess the cell viability within the proposed system, micromasses were generated within the microfluidic device and cultured under continuous perfusion of SFM enriched with 1 ng/ml TGFβ3. After three days in culture, a Live/Dead assay (Molecular Probes®, Life Technologies) was performed directly within the device, according to the manufacturer's indications. In details, a 2μM calcein AM and 4μM Ethidium homodimer-1 solution was perfused within the device for 30mins at 37°C and confocal images of labeled micromasses were subsequently acquired directly within the devices by means of a Nikon A1R Nala Confocal microscope (Nikon, Tokyo, Japan).

In Figure SI7, results relative to a representative micromass are reported and underlined a high cell viability after 3 days in culture within the device.

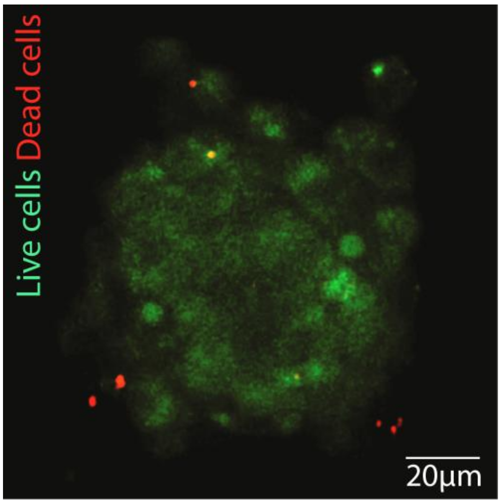

Fig.SI7 hBM-MSC microaggregates viability was assessed after three days in culture within the device under continuous perfusion of SFM enriched with 1 ng/ml TGFβ3. Results relative to a representative micromass are reported, being dead cells labeled in red, while viable ones in green.

References

[1] Martin, I., Dozin, B., Quarto, R., Cancedda, R. & Beltrame, F. 1997 Computer-based technique for cell aggregation analysis and cell aggregation in in vitro chondrogenesis. *Cytometry* **28**, 141-146. (doi:10.1002/(sici)1097-0320(19970601)28:2<141::aid-cyto7>3.0.co;2-i).
[2] ten Berge, D., Brugmann, S.A., Helms, J.A. & Nusse, R. 2008 Wnt and FGF signals interact to coordinate growth with cell fate specification during limb development. *Development* **135**, 3247-3257. (doi:10.1242/dev.023176).
[3] Weiss, S., Hennig, T., Bock, R., Steck, E. & Richter, W. 2010 Impact of growth factors and PTHrP on early and late chondrogenic differentiation of human mesenchymal stem cells. *Journal of Cellular Physiology* **223**, 84-93. (doi:10.1002/jcp.22013).
[4] Occhetta, P., Malloggi, C., Gazaneo, A., Redaelli, A., Candiani, G. & Rasponi, M. 2015 High-throughput microfluidic platform for adherent single cells non-viral gene delivery. *RSC Advances* **5**, 5087-5095. (doi:10.1039/c4ra12431f).
